# One−Step Synthesis Strategy for a Platinum−Based Alloy Catalyst Designed via Crystal−Structure Prediction

**DOI:** 10.3390/molecules29235634

**Published:** 2024-11-28

**Authors:** Dengjie Yan, Lingxin Kong, Baoqiang Xu, Bin Yang

**Affiliations:** 1 Key Laboratory for Nonferrous Vacuum Metallurgy of Yunnan Province, Kunming University of Science and Technology, Kunming 650093, China; sweams@126.com (D.Y.);; 2 National Engineering Research Center of Vacuum Metallurgy, Kunming University of Science and Technology, Kunming 650093, China; 3 Faculty of Metallurgical and Energy Engineering, Kunming University of Science and Technology, Kunming 650093, China; 4 State Key Laboratory of Complex Nonferrous Metal Resources Clean Utilization, Kunming University of Science and Technology, Kunming 650093, China

**Keywords:** crystal−structure prediction, oxygen−reduction reaction, alloy catalyst, active site, one−step preparation

## Abstract

The industrial application of polymer electrolyte membrane fuel cells is limited by the high cost of platinum catalysts. In this study, we developed a one−step synthesis strategy for low−platinum alloy catalysts based on crystal−structure predictions. Using this method, we successfully prepared a low−platinum alloy catalyst, i.e., CaPt_2_, which exhibits the same structure as its theoretically predicted counterpart in a single step via arc melting. There was no hazardous waste emission during the preparation of the alloy catalyst. Electrons were successfully enriched on the surfaces of platinum atoms, and the electronic structures of the platinum atoms were adjusted. The migration of oxygen intermediates during oxygen reduction was determined via an extensive oxygen−intermediate adsorption site test. The reaction path for the oxygen reduction process was determined. Electronic−structure analysis revealed the interaction mechanism between the oxygen intermediate and the platinum atom on the catalyst surface. The incorporation of calcium atoms into the alloy catalyst effectively improved the adsorption/dissociation state of the oxygen intermediates on the catalyst surface. Meanwhile, the molar fraction of platinum atoms in the CaPt_2_ alloy catalyst reduced by 33%, thus decreasing the feedstock cost of the catalyst. The double reduction in raw materials and manufacturing costs is conducive to the popularization and application of alloy catalysts. This study provides a reference for the design and production of other functional catalysts.

## 1. Introduction

The significant consumption of carbon−based fuels through long−term human activities has adversely affected the global climate and environment and raised concerns regarding the depleting reserves of Earth’s resources [1,2,3,4,5]. As the demand for energy increases, investigations into environmentally friendly renewable−energy sources for replacing conventional fossil fuels are becoming urgent, as is the development of clean energy−conversion technologies [1]. Hydrogen, as an energy source, offers several advantages, such as cleanliness and environmental friendliness, high levels of energy density, and renewability. Thus, it can effectively alleviate the environmental and energy issues arising from the long−term use of carbon−based fuels [6,7]. Water is the only byproduct from hydrogen production, and proton−exchange membrane fuel cells (PEMFCs), an emerging green−energy conversion technology, are expected to allow zero emissions, renewability, and high efficiency via the adoption of hydrogen energy [8,9,10]. Compared with other fuel cells, PEMFCs offer the advantages of smallness, lightness, and high fatigue resistance, which endow them with greater adaptability and reliability [11]. However, the slow kinetics of the oxygen−reduction reaction (ORR) in PEMFCs limits the wide application of clean−energy conversion technology [12,13,14,15,16]. Therefore, efficient electrocatalysts are essential for energy−conversion systems. Currently, highly active platinum group metals are used as commercial ORR catalysts, which can effectively improve the slow kinetics of ORRs [17,18,19,20,21]. However, their global scarcity and high cost limit their wide application in PEMFCs [18,19,22]. In commercial PEMFCs, the cost of platinum−based electrodes constitutes 21–45% of the total cost of fuel cells [23]. Therefore, reducing the amount of platinum in a catalyst can effectively reduce the economic cost of the catalyst. Developing platinum−free or low−platinum catalysts and improving their price competitiveness will effectively promote the large−scale application of PEMFCs [24,25,26,27].

Efforts toward the development of new catalysts have primarily focused on adjusting the electronic structures of catalysts and improving the adsorption states of the catalysts with respect to those of the intermediates [12,28,29,30]. Owing to advancements in research activities, more methods for developing new catalysts have been devised, such as doping [12,31,32], applying magnetic fields [11,33], and adjusting the spin states of active sites [4,34,35,36]. Novel catalysts, such as single−atom catalysts [37,38,39,40] and alloy catalysts [11,41,42,43,44], have been developed and exhibited high efficiency in ORRs. In single−atom catalysts, single metal atoms (SMAs) can promote the catalytic activity of the host atoms; thus, the host atoms serve as secondary active sites and have a significant synergistic effect on the catalytic performance. Although the reaction probability of the host atoms is lower than that of the SMAs, the host atoms can facilitate reductions in OER and ORR overpotentials. Wu et al. validated this by investigating the catalytic active sites of monolayer g−C_3_N_4_ (M1/g−C_3_N_4_, M = Fe, Co, Ni, Cu, and Zn) [45]. When investigating platinum−based alloy catalysts, researchers are more inclined to investigate alloy catalysts formed by platinum metal and transition metals of similar electronic configurations. Among these, transition metal alloys such as Pt−Co/Pt−Ni/Pt−Cr and Pt−Pd [41,42,46,47,48,49] have been investigated extensively. The excellent performance of binary alloys has prompted researchers to investigate multicomponent platinum−based catalysts [43,50,51], thereby further improving the efficiency of ORRs. Despite the efficient performance of alloy catalysts, their preparation process is complicated and invariably increases the manufacturing time for and economic costs of PEMFCs. The mainstream methods for the synthesis of platinum−based alloy catalysts include the polyol [50,52,53], oleylamine [54,55], impregnation−reduction [51,56,57,58], and NaBH_4_ [59,60,61] methods. However, the new structure formed after artificial intervention typically hinders the synthesis process, and the design and realization of the guide catalyst are not completely consistent.

In this study, we propose a strategy for the one−step preparation of platinum−based alloy catalysts with low platinum loadings based on a structure search and arc melting. This method involves the design and synthesis of a catalyst, which simplifies the process and improves the efficiency.

Two elements with significant electronegativity differences are more likely to form intermetallic compounds [62,63], and the corresponding preparation process is relatively simple. The crystal−structure search method is widely used in the development of new functional materials. High−performance superhard [64,65] and superconducting [66,67] materials have been successfully predicted in terms of their structures and prepared. Meanwhile, the stabilized structures achieved via a structure search can be easily prepared experimentally using a simple method. Recent studies have confirmed the key role of electron−rich metal sites in optimizing the adsorption/desorption energy barriers of oxygen reaction intermediates and accelerating the ORR pathway [68]. Due to their difference in electronegativity, platinum and calcium can form stable intermetallic compounds. Simultaneously, more electrons are enriched around the platinum atoms, which optimizes the dissociation and adsorption of the catalyst−adsorbed oxygen intermediates.

## 2. Methods

This study aims to identify Ca−Pt intermetallic compounds stabilized with different platinum contents using a structure−search method based on the Universal Structure Predictor: Evolutionary Xtallography (USPEX) [69] algorithm. We investigated the mechanism of Ca−Pt alloy catalysts and synthesized them using a simple approach. Technical details are provided in the Supporting Information.

In this study, the thermodynamic stability of Ca*_x_*Pt*_y_* structure under different stoichiometric ratios was calculated based on Formula (1).

(1) $$\begin{array}{c} ∆H=\frac{H_{{Ca}_{x}{Pt}_{y}}-xH_{Ca}-yH_{Pt}}{x+y}\; \end{array}$$

## 3. Results and Discussion

### 3.1. Structure and Stability

**Figure 1:**
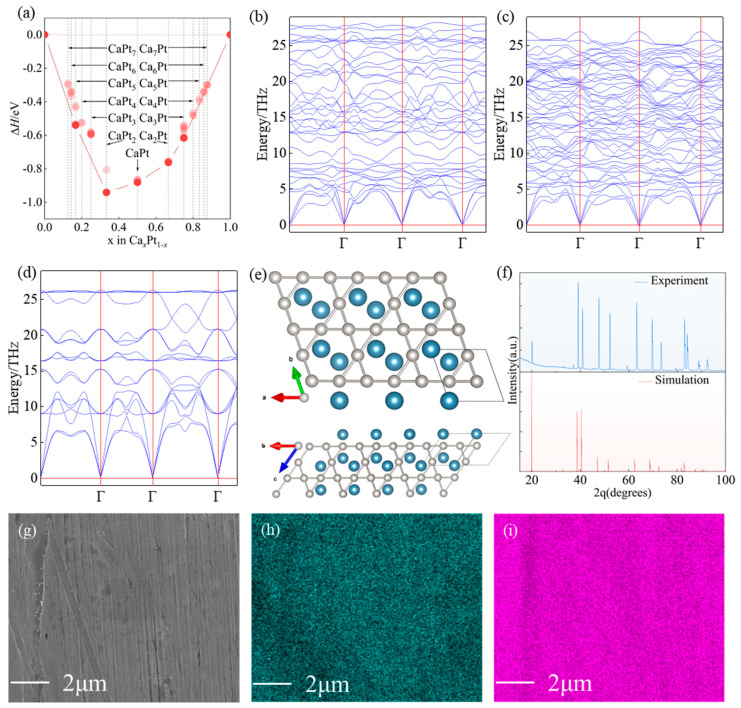
Crystal−structure prediction and experimental results. (**a**) Enthalpy of formation of stable structure predicted based on crystal structure. (**b**) Phonon dispersion of Ca_2_Pt. (**c**) Phonon dispersion of Ca_3_Pt. (**d**) Phonon dispersion of CaPt_2_. (**e**) Crystal structure of CaPt_2_, where calcium and platinum atoms are represented in blue and gray, respectively. (**f**) XRD results of CaPt_2_ structure synthesized experimentally and predicted via simulation. (**g**) SEM image of CaPt_2_. (**h**) Mapping image of Ca. (**i**) Mapping image of Pt.

Five thermodynamically stable Ca−Pt intermetallic compounds, namely, CaPt_5_(P1), CaPt_2_(*Fd−3m*), CaPt(*P63/mmc*), Ca_2_Pt(*Pnma*), and Ca_3_Pt (*Pnma*), were identified via a high−throughput search of the structure of Ca*_x_*Pt*_y_* (x:y = 1–7; x:y = 7–1), as shown in Figure 1a. However, the results of phonon−dispersion tests performed on the structures above show that the CaPt and CaPt_5_ structures were dynamically unstable (Figure 1b–d and Figure S1 in the Supporting Information). The CaPt_2_ in the stable structure exhibited a sandwich−like structure. The platinum atoms in the platinum atomic layer (001) were hexagonal, and the distance between the platinum atoms was 2.73 Å, as shown in Figure 1e. Additionally, a (010) monolayer was observed in the Ca_2_Pt structure (Figure S2a in the Supporting Information), which is composed of nonregular hexagons formed by calcium and platinum atoms. The distance between the calcium and platinum atoms was 2.92–2.97 Å. The (001) monolayer of the Ca_3_Pt structure comprised calcium and platinum atoms interspersed to form a one−dimensional chain of atoms distributed transversely. The distances between the platinum and calcium atoms on either side were 2.88 and 2.97 Å, respectively. Because the sandwich structure of Ca_2_Pt enables the platinum atoms in the catalytic environment to be utilized more effectively, the CaPt_2_ structure was selected for preparation. First, the molar ratios of different elements in the structure were converted into mass ratios. Simultaneously, considering the volatilization of calcium metal in the experimental process, an excessive amount of calcium metal is required when weighing raw materials. In this experiment, 0.907 g of platinum and 0.177 g of calcium were placed in a high−purity argon environment. A CaPt_2_ alloy was obtained by arc−melting the raw materials in an electric arc furnace. As shown in Figure 1f, the X-ray diffraction (XRD) results of the experiment and the theoretical calculations agree well. This confirms the reliability of the crystal−structure prediction results and reflects the simplicity of the alloy preparation method determined via structure prediction.

Meanwhile, it can also be observed that there is a certain degree of peak intensity mismatch in the XRD pattern, which implies that there exist certain orientation differences and lattice distortions in the CaPt_2_ alloy. During the arc−melting process, materials undergo rapid heating and cooling cycles, causing thermal stresses as the temperature changes rapidly. These thermal stresses can affect the growth orientation of crystals, resulting in certain orientation differences in the growth process of crystals in different regions. Additionally, the fast cooling rate after arc−melting leads to rapid solidification, during which atoms may be “frozen” in non−equilibrium positions, creating defects like vacancies and interstitial atoms that affect the surrounding lattice and cause distortion. These orientation differences may have a certain impact on catalytic activity. Different orientations can affect the distribution and accessibility of the active sites on the catalyst surface. For example, a specific orientation may make the active sites more favorable for the adsorption of reactant molecules, while other orientations may hinder the approach of reactants to some extent, thus changing the initial steps and rate of the catalytic reaction. This point deserves more in−depth and systematic research.

The SEM/EDS results are shown in Figure 1g–i. The SEM results indicate that the alloy has a uniform texture, and the calcium and platinum elements are evenly distributed throughout the observed area. Their atomic ratio is highly consistent with the expected composition of CaPt_2_, further confirming the successful preparation of the target alloy.

### 3.2. Electronic Properties of CaPt_2_

**Figure 2:**
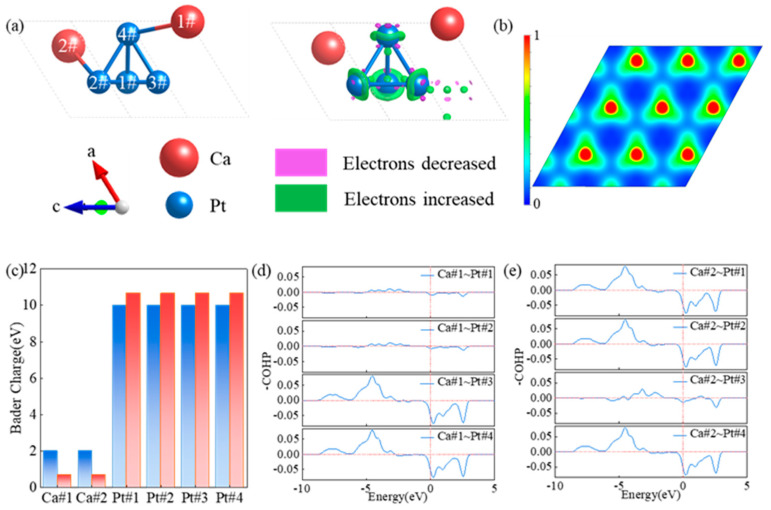
Charge characteristics of calcium and platinum atoms in CaPt_2_ structure. (**a**) Differential charge density of CaPt_2_ structure. (**b**) Electron localization function of CaPt_2_ structure. (**c**) Charge gain and loss of calcium and platinum atoms in CaPt_2_ structure. The number of electrons is indicated in blue for before alloying and red for after alloying. (**d**,**e**) Bonding characteristics between calcium and platinum atoms in CaPt_2_ structure.

The results of differential charge−density analysis (Figure 2a) show that the charge density around the platinum atoms in the direction of the line connecting the calcium and platinum atoms in the CaPt_2_ structure increased. Electron enrichment was successfully achieved around these atoms. The crystal orbital Hamiltonian preoccupation (COHP) can be used to determine whether atoms are bonded. Below the Fermi energy, two atoms with a COHP less than 0 are in the bonded state, whereas those with a COHP exceeding 0 are in the antibonded state. Both the calcium and neighboring platinum atoms exhibited effective bonding states below the Fermi energy (Figure 2d–e). However, if the distance between atoms is extremely large or an interval atom exists in the atomic connection, then the bonding state of the atoms will be affected significantly. The distances of atom Ca#1 from atoms Pt#1 and Pt#2 as well as the distance of atom Ca#2 from atom Pt#3 atom in the CaPt_2_ structure exceeded 5 Å, which resulted in weak interatomic interactions. The COHPs between the atoms of the same species in the CaPt_2_ structure (Figure S3 in the Supporting Information) show different bonding trends at different energies below the Fermi energy. The calcium atoms exhibited localized antibonding states at energy levels of less than −5 eV, whereas they exhibited delocalized bonding states at energy levels above −5 eV. The −COHP integral value between the calcium atoms below the Fermi level was only 0.002 eV, thus indicating that the overall interaction between the calcium atoms was negligible. The COHP of the platinum atoms exhibited an opposite tendency to that of the calcium atoms; i.e., the platinum atoms exhibited bonding states at energy levels below −3 eV, and vice versa for antibonding states. The −COHP integral value between the platinum atoms below the Fermi energy were approximately 0.55 eV, thus indicating a certain weak interaction between different platinum atoms. The electron localization function (ELF) reveals the distribution of electrons in space, particularly the localization degree of electron pairs. In fact, the ELF can be used to analyze the bonding properties between atoms. An ELF value of less than 0.5 signifies an ionic bond, whereas an ELF value exceeding 0.5 signifies a covalent bond. Figure 2b shows the ELF of the 001 plane of the CaPt_2_ structure, and the lattice−plane position of the ELF is shown in Figure S4 (Supporting information). The charge in space was primarily localized around the platinum atoms. Meanwhile, the ELF value between the platinum and calcium atoms was significantly lower than 0.5. Therefore, the bonding between the platinum and calcium atoms showed a strong ionic form. The results of differential charge density and ELF analysis showed that an ionic bond with the charge around the platinum atom was formed between the calcium and platinum atoms. Additionally, the results of Bader charge analysis (Figure 2c) indicate that the platinum atoms gained electrons and the calcium atoms lost electrons during the bonding process. During the bonding process, each calcium atom lost an average of 1.31 electrons and was evenly distributed with respect to each platinum atom, thereby revealing a clear negative oxidation state of the platinum atoms in the alloy catalyst.

### 3.3. Action Mechanism of CaPt_2_ and Oxygen Intermediates

**Figure 3:**
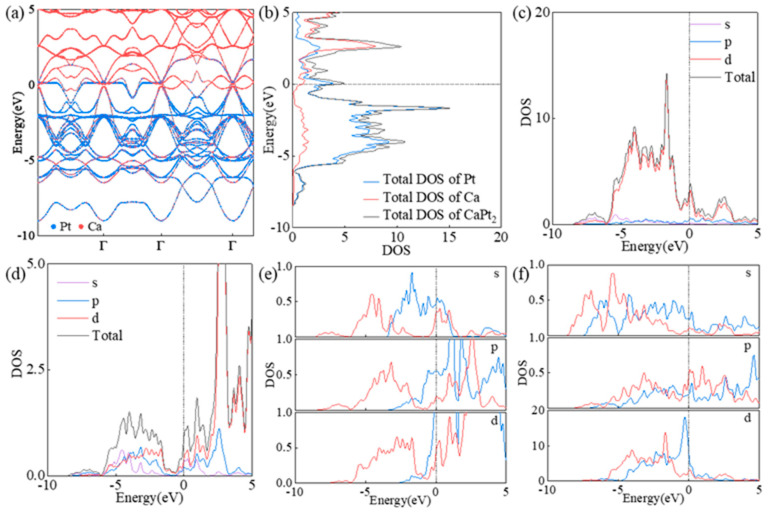
Electronic structure of CaPt_2_. (**a**) Energy band structure of CaPt_2_. (**b**) Total density of states of CaPt_2_ structure and projected density of states of calcium and platinum atoms. (**c**) Orbital projected density of states of platinum atoms in CaPt_2_ structure. (**d**) Orbital projected density of states of calcium atoms in CaPt_2_ structure. (**e**,**f**) Comparison of orbital projected density of states of calcium and platinum atoms in CaPt_2_ structure and comparison model. Orbital−projected state densities of calcium and platinum atoms in CaPt_2_ structure are shown in red, and orbital−projected state densities in comparison models are shown in blue.

The performance of catalysts is significantly affected by their electronic structures [28]. The projected band structure and density of states (Figure 3a–c) analysis of CaPt_2_ show that some electrons were active at the Fermi energy, which is conducive to the electron crosslinking between the alloy catalyst and adsorption intermediates. Before the interaction between the oxygen molecule and catalyst, the valence−electron number of calcium was two, and all electrons were located in the 4s orbital. However, in the CaPt_2_ alloy catalyst, the density of states of calcium atoms shows the contribution of p− and d−orbitals below the Fermi energy (Figure 3d), thus indicating the electronic structures of transition metals. To determine the source of the changes in the electronic structures of the calcium atoms, we constructed two comparison models (Figure S5 in the Supporting Information). The models were based on the original CaPt_2_ cell structure, which were constructed by removing one atom type from the cell and retaining the other atom type. An analysis of the density of states of calcium atoms in the alloy catalyst and the comparison model (Figure 3e) shows that platinum atoms caused the electronic state of the calcium atoms to change to a transition−metal−like state. For the fully occupied s−orbital, the electron shifted to a position with an extremely low energy level, and the electron distribution was more localized. In the empty p−orbital of the calcium atoms, an electron distribution was clearly observed below the Fermi energy, and a certain local state was exhibited. The local state of the calcium atoms’ p−orbital was primarily contributed by the p_x_−orbital (Figure S6b in the Supporting Information), and the p_y_− and p_z_−orbitals were primarily delocalized. Meanwhile, the d−orbital of the calcium atoms shows the distribution of electronic states (Figure S6c in the Supporting Information), and the energy position of the distribution was similar to that of the p−orbital. However, the electronic states of the d−orbital primarily exhibited delocalized states, and the electronic states at each energy position were uniformly contributed by the suborbitals of the d−orbital. In the CaPt_2_ alloy catalysts, the electronic structures of the calcium and platinum atoms showed reciprocal effects. Owing to the introduction of calcium atoms in the comparative model, the density of electronic states in all orbitals of the platinum atoms shifted to lower energies. The electronic state of the platinum atoms’ s−orbital near the Fermi energy declined significantly (Figure 3f), and the electron distribution was more delocalized. The occupancy of the p−orbital electrons of the platinum atoms did not change significantly; however, a certain degree of peak splitting occurred. This phenomenon was primarily caused by the electron localization of the p_y_− and p_z_−orbitals (Figure S7b in the Supporting Information). Meanwhile, the d−orbital of the platinum atoms, which contribute prominently to the catalytic function, showed peak splitting (Figure S7c in Supporting Information), thereby facilitating the interaction of the alloy catalysts with the oxygen intermediates.

**Figure 4:**
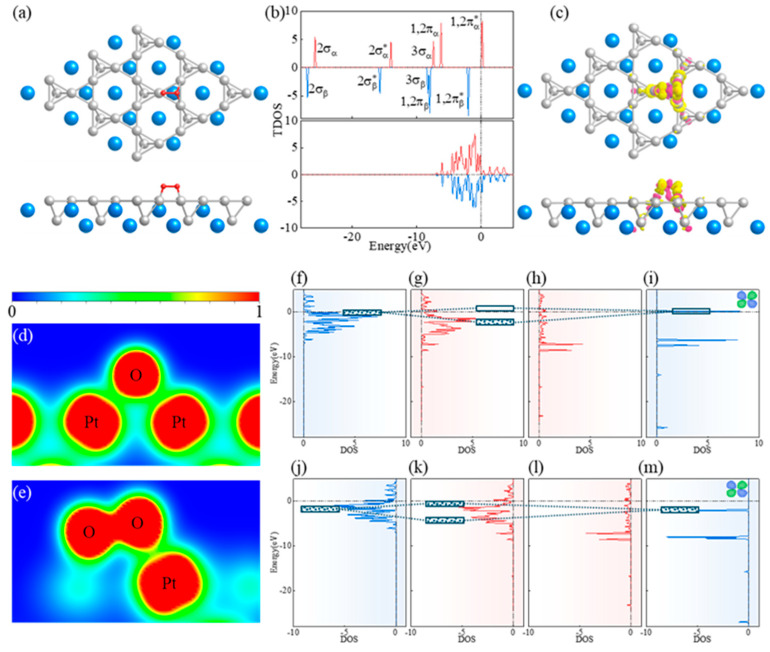
Mechanism by which an oxygen molecule is adsorbed on CaPt_2_ alloy catalyst. (**a**) Stable adsorption configuration of oxygen molecule on CaPt_2_ catalyst. (**b**) Density of states of oxygen molecule and projected density of states of d−orbitals of platinum atoms before oxygen−molecule adsorption on catalyst. (**c**) Differential charge density of oxygen molecule adsorbed onto CaPt_2_ alloy catalyst. (**d**,**e**) electron localization function of oxygen−molecule adsorption onto catalyst at different angles. (**f**–**i**) Interaction of spin−up electrons when oxygen molecule was adsorbed onto catalyst. (**j**–**m**) Interaction of spin−down electrons when oxygen molecule was adsorbed onto catalyst.

In order to examine the performance of the alloy catalyst, a slab model was constructed based on the 001 surface of the CaPt_2_ alloy catalyst (Figure S8 in the Supporting Information). The ORR begins with the adsorption of oxygen molecules. To identify the adsorption sites of oxygen molecules on the catalyst, nine possible adsorption models of oxygen molecules on the catalyst were established and named #1–#9 (Figure S9 in the Supporting Information). After performing structural optimization on the models above, the oxygen molecules in Models #1, #2, #3, and #9 could not be stabilized at the initial position (Figure S10 in the Supporting Information). In Models #3 and #9, the oxygen molecule was stabilized at the hollow position in the form of a flat layer, which is consistent with the configuration of the lowest energy structure in the stable model. Considering stability and energy (Figure S11 in the Supporting Information), Model #7 (Figure 4a) was selected as the adsorption model for oxygen molecules on the alloy catalyst.

During adsorption, the oxygen molecule primarily interacted with the d−orbital of platinum in the catalyst near the Fermi energy. As a high−spin gas molecule, oxygen has a 1,2π* antibonding orbital in both the spin−up and spin−down states, which is the closest to the Fermi energy (Figure 4b). Therefore, the interaction between the oxygen molecule and catalyst was primarily composed of the 1,2π* antibonding orbital and the d−orbital of the platinum atoms. The charge transfer of the oxygen molecules before and after adsorption on the catalyst was determined by performing a differential−charge−density analysis (Figure 4c). During adsorption, electrons were transferred from the platinum atoms to the oxygen atoms. Simultaneously, the electronic layout inside the oxygen molecule changed. Unadsorbed oxygen molecules accumulated numerous electrons between two oxygen atoms to form covalent bonds. After the oxygen molecules were adsorbed onto the CaPt_2_ alloy catalyst, some of the electrons used to form covalent bonds between the oxygen atoms were transferred between the oxygen and platinum atoms to form Pt−O covalent bonds (Figure 4d). However, no bond breaking occurred between the oxygen atoms, and the oxygen atoms maintained relatively weak covalent bonds (Figure 4e). For the α−orbital, the 1,2π_α_* antibonding orbital of the oxygen molecule (Figure S12 in the Supporting Information) interacted with the d−orbital of the platinum atoms, and the mode of this action was the interaction between the non−occupied orbital (HOMO) and the occupied orbital. The $d_{z^{2}}$− and $d_{x^{2}-y^{2}}$−orbitals of the platinum atoms contributed significantly to the interaction with the oxygen atoms (Figure S13 in the Supporting Information). As shown in Figure 4f–i, the interaction between the occupied and unoccupied orbitals yielded a bonding orbital with a lower energy level and an antibonding orbital with a higher energy level (above the Fermi energy), which is conducive to the reduction in the overall energy. In the β−orbital, the 1,2π_β_* antibonding orbital of the oxygen molecule (Figure S14 in the Supporting Information) interacted with the d−orbital of the platinum atoms, and the interaction mode with the catalyst surface was the interaction between the non−occupied orbital (LOMO) and occupied orbital. Among them, the d_xz_− and d_yz_−orbitals of the platinum atoms contributed significantly to the interactions (Figure S15 in the Supporting Information). The bonding and antibonding orbitals generated by this mode of action were located below the Fermi energy (Figure 4j–m), which is theoretically unfavorable for the overall energy. However, Figure 4j,k show that more empty states appeared above the Fermi energy after the adsorption of oxygen molecules. This is owing to the reflux of electrons into the inner layer of the catalyst, which is conducive to the reduction in the overall energy.

**Figure 5:**
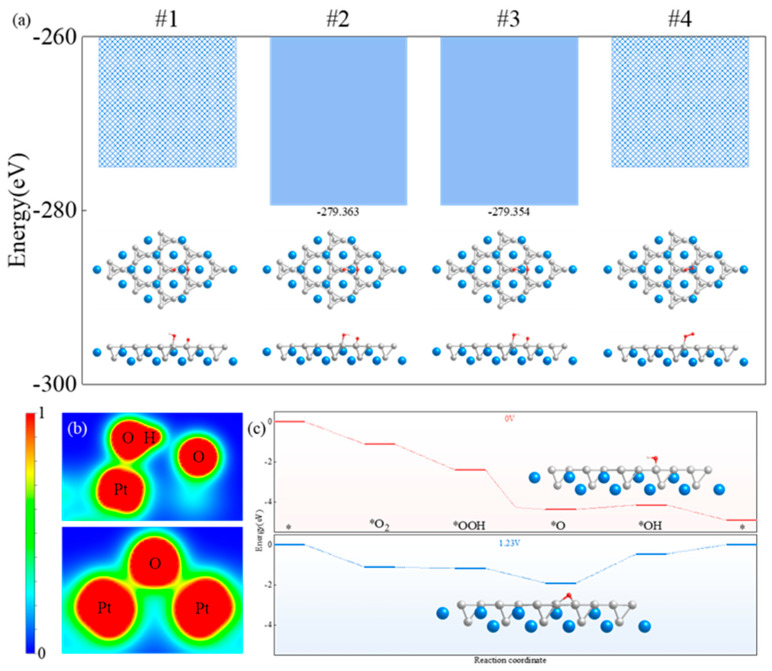
OOH−adsorption−site test process and energy changes of each intermediate state during ORR. (**a**) Energy of OOH intermediates with different configurations adsorbed onto catalyst. (**b**) Electron localization functions of OOH adsorbed on catalyst at different angles. (**c**) Energy changes during ORR.

The hydrogenation of the oxygen molecules adsorbed onto the catalyst showed different configurations. Based on the different hydrogenation positions, four *OOH models (Figure S16 in the Supporting Information) were constructed and named #1–#4. After structural optimization, the #1 and #4 configurations were unstable, and the #2 configuration showed the lowest energy (Figure 5a). Therefore, #2 was selected as the adsorption configuration for the OOH intermediates. The hydrogen atom entered the adsorption group, and the covalent bond between the oxygen atoms was broken. The top−site oxygen atom combined with the hydrogen atom to form a covalent bond, whereas the platinum and oxygen atoms remained covalently bonded to each other (Figure 5b).

Next, the two− and four−electron processes of the ORR were examined. The two−electron ORR process directly generating H_2_O_2_ was not feasible, and the reaction path was a single four−electron process. After *OOH was further hydrogenated, a water molecule was formed and desorbed, thus resulting in an oxygen atom on the alloy catalyst. The configurations of the oxygen atom and the molecule are different, and this can lead to the differences in adsorption sites and adsorption characteristics. After the desorption of the water molecule, the adsorption sites of the oxygen atoms on the catalyst may shift within a small range. In this study, six adsorption models for oxygen atoms on the catalyst (Figure S17 in the Supporting Information) were constructed (named #1–#6) and tested. The oxygen atoms located at different adsorption sites shifted to adjacent hollow sites during the structural−optimization process. Optimizing the six different configurations of the oxygen−atom adsorption models resulted in all of the oxygen atoms appearing at the hollow site. The energy of the oxygen atom at the hollow site directly above the calcium atom was lower than that at the hollow site directly above the platinum atom. Therefore, the adsorption site of the oxygen atom was determined to be at the hollow site directly above the calcium atom (Figure S18 in the Supporting Information). One can infer that after the hydrogenation of *OH at the top site, another oxygen atom at the bridge site shifted to the hollow site. After determining the adsorption site of a single oxygen atom, the path of the entire ORR was clarified. However, *O typically presents an energy barrier to hydrogenation. Based on the angle between the hydrogen and oxygen atoms, five different *OH adsorption models were established and named #1–#5 (Figure S19 in the Supporting Information). Repulsion was indicated between the hydrogen and platinum atoms, which caused the hydrogen atom to deviate from the platinum atom to the calcium atom. The strong covalent bond between the hydrogen and oxygen atoms caused the oxygen atom to shift to the bridge site during the migration of the hydrogen atom. However, in the #5 *OH adsorption model, the hydrogen atom was distant from the platinum atom, and the oxygen atom hindered the interaction between the hydrogen and platinum atoms to a certain extent. Therefore, the position of the oxygen atom in configuration #5 did not change significantly after the adsorption of the hydrogen atoms. Based on the energy of the adsorption configuration, #1 was determined to be the optimal configuration for OH adsorption (Figure S20 in the Supporting Information). In this study, we classified the ORR into five steps. The first three elementary reaction steps were determined to be completely exothermic (Figure 5c), which effectively improves the ORR kinetics. The reaction from *O to *OH during the ORR was determined to be the rate−determining step.

## 4. Conclusions

In this study, we successfully designed and synthesized an alloy catalyst. Stable CaPt_2_, Ca_2_Pt, and Ca_3_Pt structures were successfully obtained via crystal−structure prediction. The CaPt_2_ alloy catalyst was prepared in a single step via arc melting. There was no hazardous waste emission during the preparation of the alloy catalyst. The result of electronic−structure analysis revealed that the addition of calcium atoms successfully increased the number of electrons on the surface of the catalyst, thus improving the adsorption of the catalyst. Additionally, the optimal adsorption site, adsorption mechanism, and reaction path were determined by investigating the adsorption of oxygen intermediates on the catalyst. Finally, the molar fraction of platinum reduced by 33% in the CaPt_2_ catalyst designed and synthesized using this strategy. In conclusion, the proposed strategy effectively reduces the cost of raw materials and manufacturing and overcomes the barrier to the popularization and application of platinum−based catalysts. It is expected to promote the application of platinum−based catalysts in industrial and commercial fields.

## Data Availability

The data used in the article are included in the main text and its Supporting Information.

## Supplementary Materials

The following supporting information can be downloaded at: https://www.mdpi.com/article/10.3390/molecules29235634/s1, Table S1: The energy and force convergence criteria for each step; Table S2: Crystal structure parameters of Ca-Pt intermetallic compounds; Figure S1: Phonon dispersion of dynamically unstable structure. (a) CaPt. (b) CaPt_5_; Figure S2: Crystal structures. (a) Ca_2_Pt. (b) Ca_3_Pt; Figure S3: The COHPs between the atoms of the same species in the CaPt_2_ structure. (a) COHP values between Ca atoms. (b,c) COHP values between Pt atoms; Figure S4: Lattice-plane position; Figure S5: Original and contrasting structure models. (a) Original structure. (b) Calcium contrasting structure model. (c) Platinum contrasting structure model; Figure S6: Comparison of orbital projected density of states of calcium atoms in CaPt_2_ structure and comparison model. (a) s orbital. (b) p orbital. (c) d orbital. Orbital-projected state densities of calcium atoms in CaPt_2_ structure are shown in red, and orbital-projected state densities in comparison model are shown in blue; Figure S7: Comparison of orbital projected density of states of platinum atoms in CaPt_2_ structure and comparison model. (a) s orbital. (b) p orbital. (c) d orbital. Orbital-projected state densities of platinum atoms in CaPt_2_ structure are shown in red, and orbital-projected state densities in comparison model are shown in blue; Figure S8: Slab model based on CaPt_2_ (001) surface; Figure S9: Possible oxygen adsorption configurations on the catalyst. (a) to (i) represent Model #1 to Model #9; Figure S10: Configurations after oxygen adsorption on the catalyst. (a) to (i) represent Model #1 to Model #9; Figure S11: Energies of different configurations of oxygen after adsorption on the catalyst; Figure S12: Oxygen molecular orbital model (spin-up); Figure S13: Density of projected states of the d orbital (spin-up) of platinum atoms on the catalyst surface before oxygen adsorption; Figure S14: Oxygen molecular orbital model (spin-down); Figure S15: Density of projected states of the d orbital (spin-down) of platinum atoms on the catalyst surface before oxygen adsorption; Figure S16: Possible OOH adsorption configurations on the catalyst. (a) to (d) represent Model #1 to Model #4; Figure S17: Possible O adsorption configurations on the catalyst. (a) to (e) represent Model #1 to Model #6; Figure S18: Energies of the O atom adsorbed at different sites on the catalyst; Figure S19: Possible OH adsorption configurations on the catalyst. (a) to (e) represent Model #1 to Model #5; Figure S20: Energies of different configurations of OH after adsorption on the catalyst.
