# Supplementary material for: One−Step Synthesis Strategy for a Platinum−Based Alloy Catalyst Designed via Crystal−Structure Prediction
Source: Molecules. 2024 Nov 28;29(23):5634. doi: 10.3390/molecules29235634 (PMC11642978; doi:10.3390/molecules29235634)
Supplement: Supplementary file 1 [file molecules-29-05634-s001.zip › molecules-3289367-supplementary.pdf]

One-step synthesis strategy of platinum-based alloy catalyst designed via crystal-structure prediction

Dengjie Yan [1,2,3], Lingxin Kong [1,2,3,4*], Baoqiang Xu [1,2,3,4], Bin Yang [1,2,3,4*]

[1] Key Laboratory for Nonferrous Vacuum Metallurgy of Yunnan Province, Kunming University of Science and Technology, Kunming, 650093, China

[2] Faculty of Metallurgical and Energy Engineering, Kunming University of Science and Technology, Kunming, 650093, China

[3] National Engineering Research Center of Vacuum Metallurgy, Kunming University of Science and Technology, Kunming, 650093, China

[4] State Key Laboratory of Complex Nonferrous Metal Resources Clean Utilization, Kunming University of Science and Technology, Kunming, 650093, China

*. Corresponding author. E-mail address: konglx@kust.edu.cn (L.X. Kong); kgyb2013@kust.edu.cn (B. Yang)

Computational Details

The crystal structure prediction of the Ca-Pt alloy catalyst was performed by USPEX (Universal Structure Predictor: Evolutionary Xtallography) and VASP (Vienna Ab initio Simulation Package) softwares. The thermodynamically stable crystal structures of Ca_xPt_y (x:y = 1-7; x:y = 7-1) were predicted at 0 GPa pressure. In the first step, 30 random structures with certain symmetry were constructed in which atomic coordinates were generated by the crystallographic symmetry operations. A 6-step hierarchical optimization strategy was used in the optimization process of each structure. ISIF = 4 was used in the structural optimization of steps 1 and 2, and ISIF = 3 was used in the optimization of steps 3-6. The energy and force convergence criteria for each step are shown in Table S1.

Table S1. The energy and force convergence criteria for each step.

Step	EDIFF	EDIFFG
1	$2*10^{-3}$	$-2*10^{-1}$
2	$1*10^{-3}$	$1*10^{-2}$
3	$1*10^{-3}$	$1*10^{-2}$
4	$1*10^{-4}$	$1*10^{-3}$
5	$1*10^{-5}$	
6	$3*10^{-6}$	

Starting from generation 2, 20 structures were generated per generation. Based on the processed structures from the previous step, 50 % of the 20 structures were produced by heredity, 30 % by randomly from space groups, and 20 % by softmutation. When no new low-energy structure was produced for 10 consecutive generations, it was considered that the ground state structure has been successfully searched and the crystal structure search process was judged to be over.

More accurate structural optimizations were conducted using the VASP code for low-energy structures generated by structural prediction. The cut-off energy for the expansion of wavefunctions into plane waves was set to 250 eV in all calculations. K-Spacing value to generate K-Mesh was set to 0.02$Å^{-1}$. Electron-ion interactions were described within the projector augmented wave method with s2d0 and s1d9 orbitals as valence states for Ca and Pt atoms, respectively.

Table S2. Crystal structure parameters of Ca-Pt intermetallic compounds.

	Space group	Lattice parameters		Atom	Atomic positions (Fractional coordinates)		
Ca_3Pt	Pnma	a = 7.74358	α = 90.0919	Ca	0.83229	0.56429	0.33444
		b = 9.62248	β = 89.9205	Ca	0.33215	0.93297	0.16049
		c = 6.66217	γ = 89.9453	Ca	0.66926	0.06534	0.83368
				Ca	0.16930	0.43399	0.66064
				Ca	0.83096	0.93283	0.33485
				Ca	0.33031	0.56467	0.15920
				Ca	0.67114	0.43365	0.83559
				Ca	0.17070	0.06543	0.65915
				Ca	0.04176	0.24744	0.14093
				Ca	0.54140	0.25025	0.35368
				Ca	0.46035	0.74810	0.64031
				Ca	0.95957	0.75050	0.85400
				Pt	0.37677	0.25074	0.95141
				Pt	0.87653	0.25015	0.54244
				Pt	0.12506	0.74862	0.45229
				Pt	0.62476	0.74879	0.04285
Ca_2Pt	Pnma	a = 9.24450	α = 89.9950	Ca	0.57121	0.49994	0.39257
		b = 4.42757	β = 90.1163	Ca	0.07040	0.00535	0.59938
		c = 7.85556	γ = 89.8218	Ca	0.43147	0.99961	0.10467
				Ca	0.93206	0.50249	0.88752
				Ca	0.66505	0.99769	0.72435
				Ca	0.16601	0.50559	0.27000
				Ca	0.33433	0.50028	0.77295
				Ca	0.83447	0.00477	0.21749
				Pt	0.12397	0.00259	0.98789
				Pt	0.62385	0.49697	0.00350
				Pt	0.88027	0.50941	0.49880
				Pt	0.37952	0.99965	0.49231
CaPt	P63/mmc	a = 4.54734	α = 89.9981	Ca	0.66998	0.34349	0.99563
		b = 4.55521	β = 89.9912	Ca	0.67181	0.33619	0.49424

Table S2(continued). Crystal structure parameters of Ca-Pt intermetallic compounds.

	Space group	Lattice parameters		Atom	Atomic positions (Fractional coordinates)		
		c = 11.08687	γ = 119.9245	Ca	0.33671	0.66685	0.74471
				Ca	0.33444	0.67429	0.24423
				Pt	0.00169	0.01115	0.11931
				Pt	0.00542	0.00025	0.61917
				Pt	0.00233	0.00432	0.36916
				Pt	0.00462	0.00435	0.86946
$CaPt_2$	Fd-3m	a = 5.45758	α = 60.0267	Ca	0.99354	0.01290	0.98247
		b = 5.45825	β = 119.941	Ca	0.74146	0.76313	0.48264
		c = 5.45841	γ = 89.9485	Pt	0.36490	0.88832	0.73095
				Pt	0.36550	0.38819	0.73093
				Pt	0.36415	0.38824	0.23048
				Pt	0.86452	0.38843	0.23142
$CaPt_5$	P1	a = 9.33372	α = 89.9896	Ca	0.99895	0.99763	0.44121
		b = 9.33574	β = 89.9611	Ca	0.66650	0.33118	0.47235
		c = 4.44151	γ = 119.973	Ca	0.33525	0.66810	0.48287
				Pt	0.00011	0.49843	0.96829
				Pt	0.50105	0.99920	0.96553
				Pt	0.50208	0.49785	0.96904
				Pt	0.66839	0.00032	0.46421
				Pt	0.66672	0.66341	0.46688
				Pt	0.00089	0.66454	0.46669
				Pt	0.33243	0.99986	0.46497
				Pt	0.33268	0.33274	0.46681
				Pt	0.00259	0.33326	0.46617
				Pt	0.66602	0.83118	0.96494
				Pt	0.83547	0.66748	0.96578
				Pt	0.16819	0.83207	0.96549
				Pt	0.33484	0.16596	0.96439
				Pt	0.16821	0.33292	0.96649
				Pt	0.83495	0.16496	0.96331

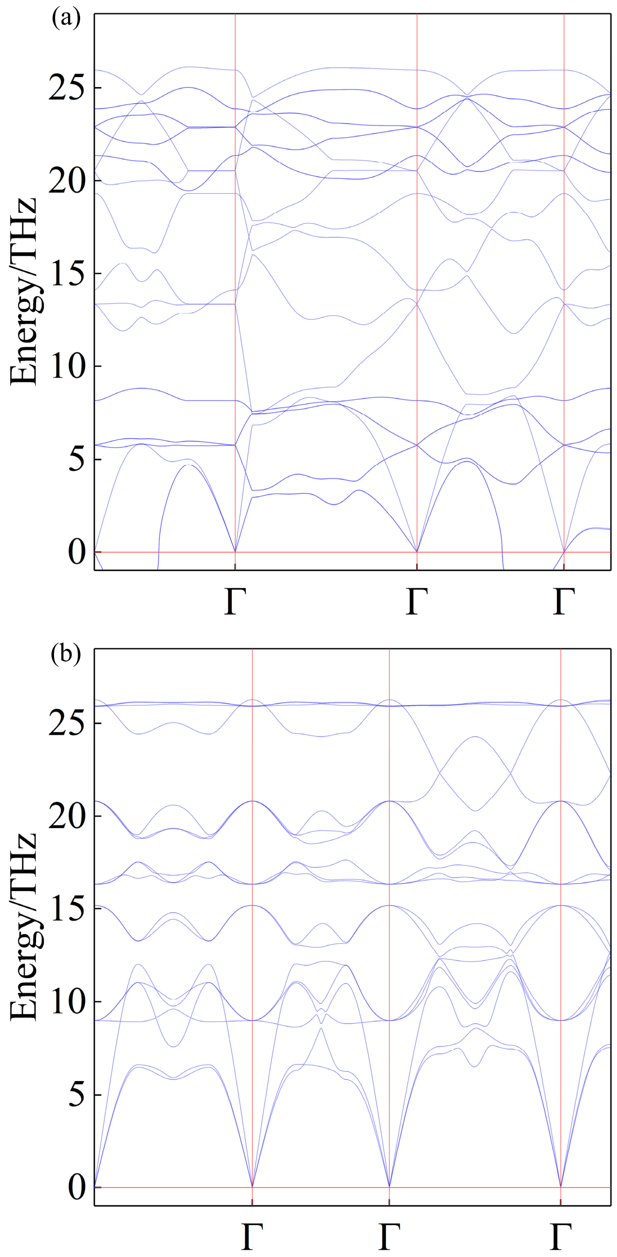

Fig. S1. Phonon dispersion of dynamically unstable structure. (a) CaPt. (b) $CaPt_5$.

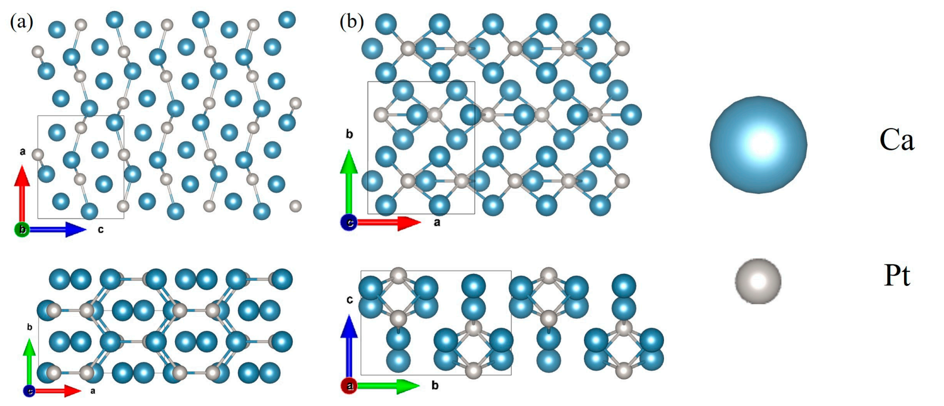

Fig. 2. Crystal structures. (a) Ca_2Pt. (b) Ca_3Pt.

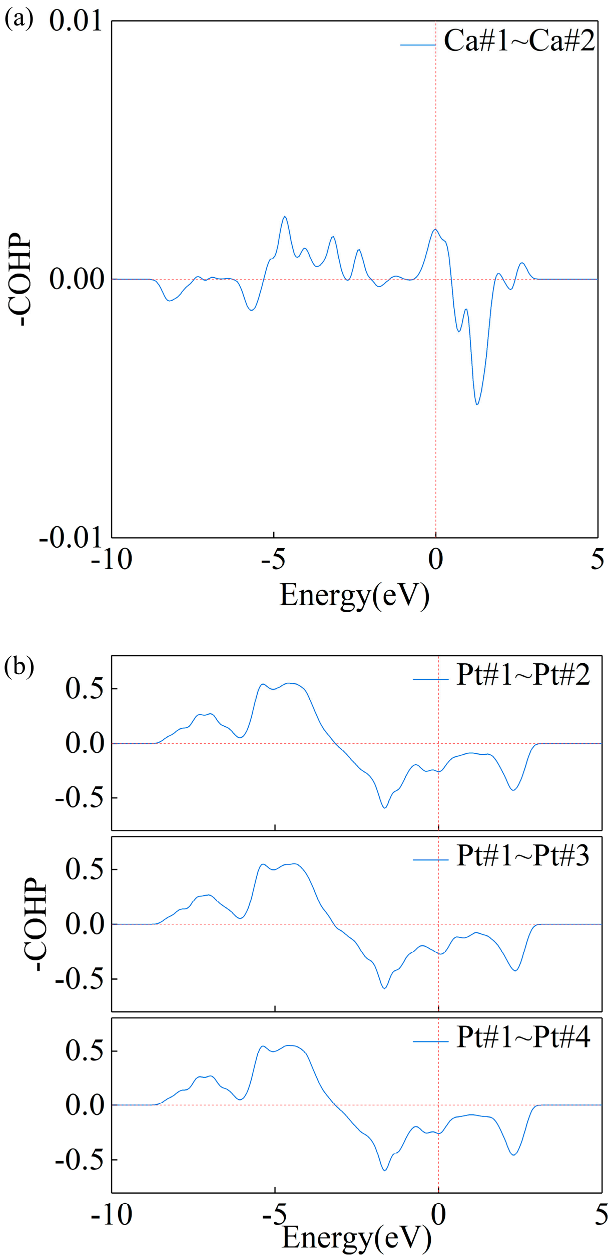
(a)
0.01
0.00
-0.01
-COHP
Ca#1~Ca#2
-10
-5
0
5
Energy(eV)
(b)
Pt#1~Pt#2
Pt#1~Pt#3
Pt#1~Pt#4
0.5
0.0
-0.5
-COHP
-10
-5
0
5
Energy(eV)

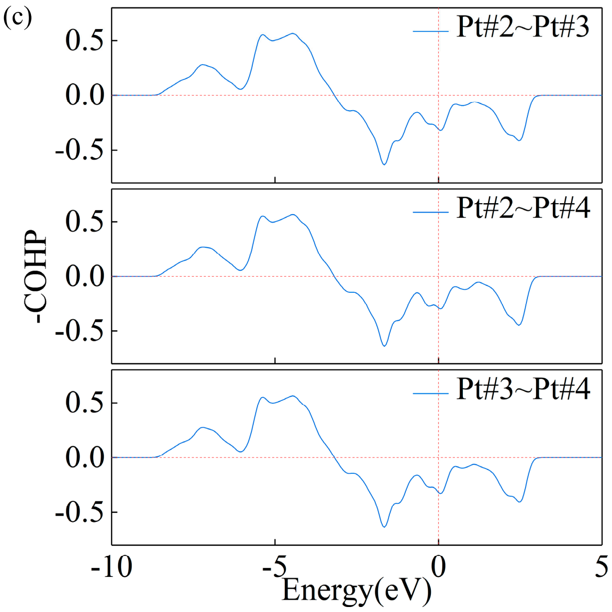

Fig. S3. The COHPs between the atoms of the same species in the $CaPt_2$ structure. (a) COHP values between Ca atoms. (b-c) COHP values between Pt atoms.

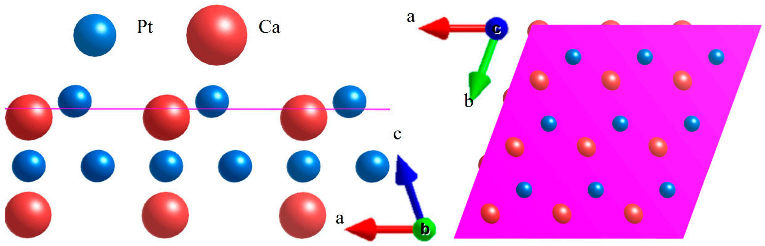

Fig. S4. Lattice-plane position.

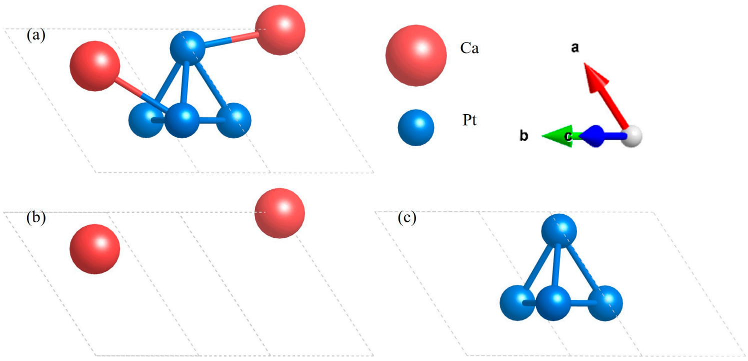

Fig. S5. Original and contrasting structure models. (a) Original structure. (b) Calcium contrasting structure model. (c) Platinum contrasting structure model.

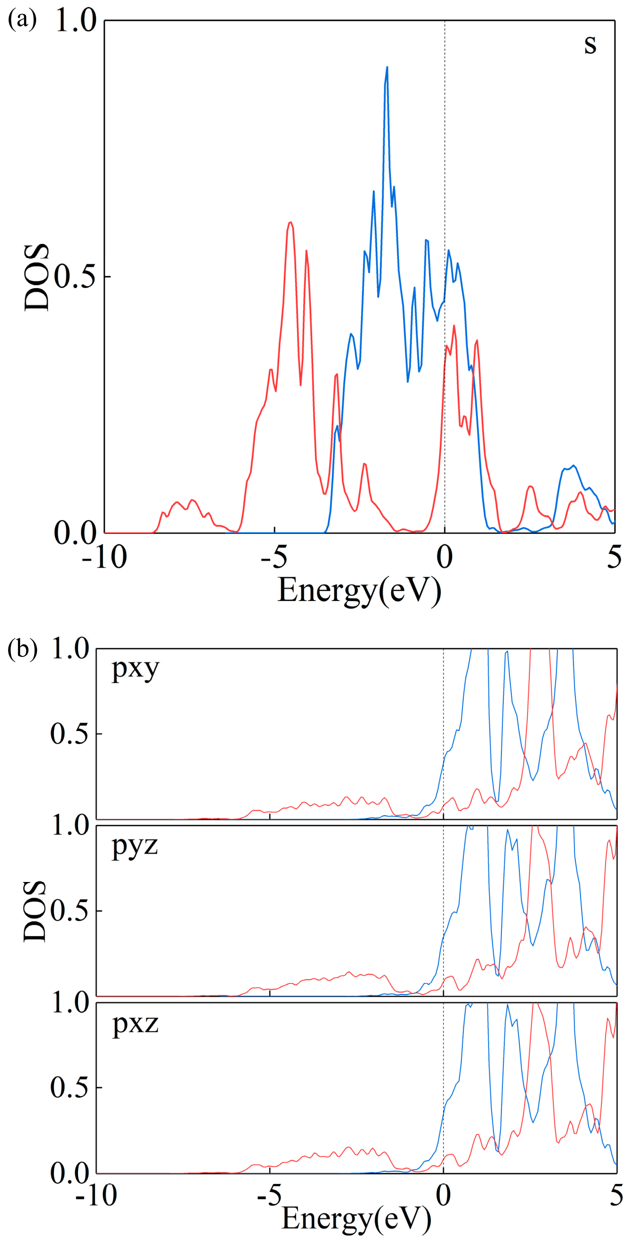

(a)
s
DOS
1.0
0.5
0.0
-10
-5
0
5
Energy(eV)
(b)
pxy
pyz
pxz
DOS
1.0
0.5
0.0
-10
-5
0
5
Energy(eV)

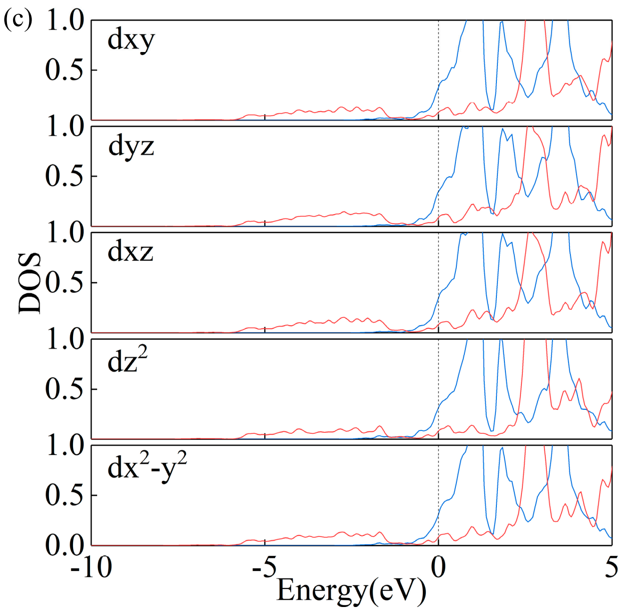

Fig. S6. Comparison of orbital projected density of states of calcium atoms in $CaPt_2$ structure and comparison model. (a) s orbital. (b) p orbital. (c) d orbital. Orbital-projected state densities of calcium atoms in $CaPt_2$ structure are shown in red, and orbital-projected state densities in comparison model are shown in blue.

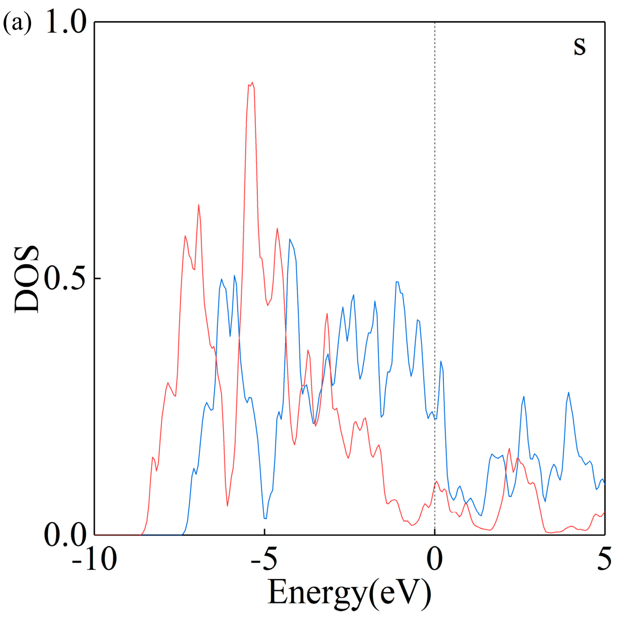

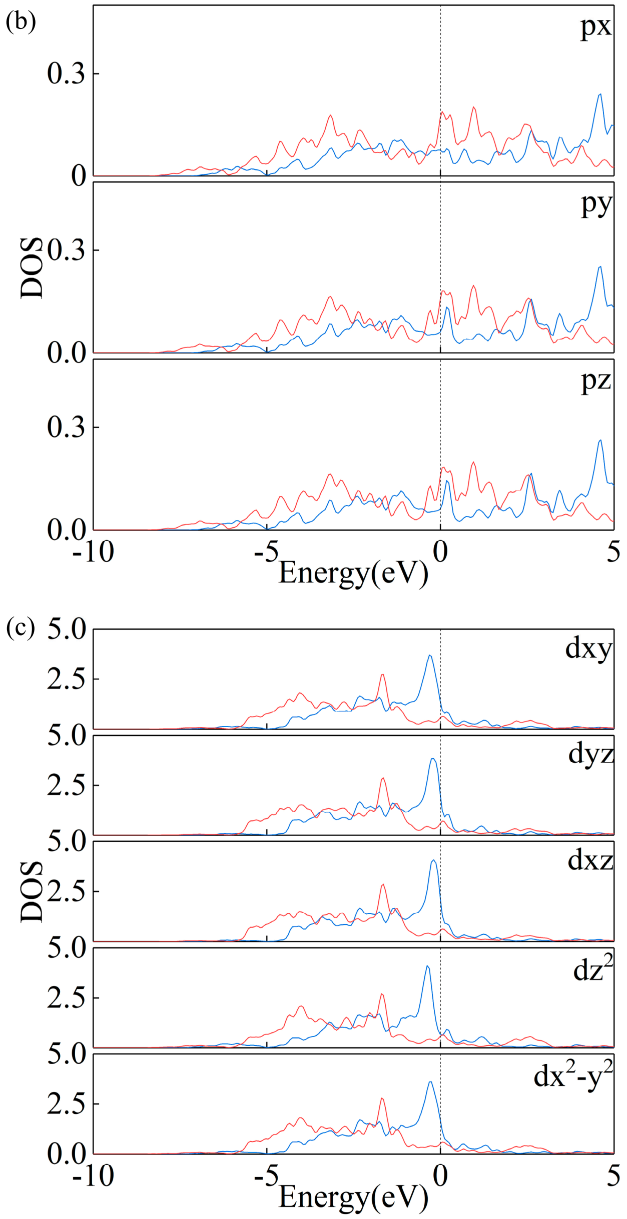

Fig. S7. Comparison of orbital projected density of states of platinum atoms in $CaPt_2$ structure and comparison model. (a) s orbital. (b) p orbital. (c) d orbital. Orbital-projected state densities of platinum atoms in $CaPt_2$ structure are shown in red, and orbital-projected state densities in comparison model are shown in blue.

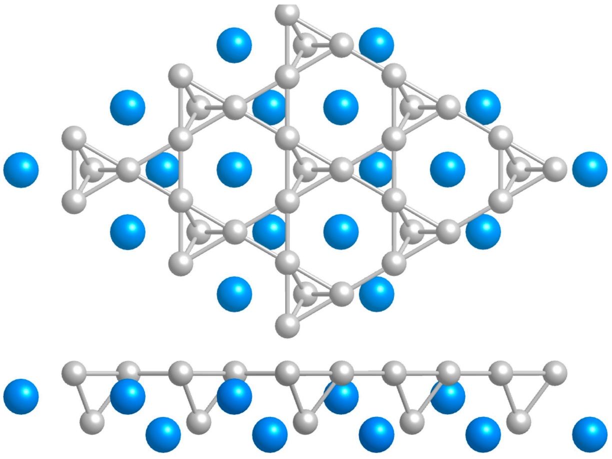

Fig. S8. Slab model based on $CaPt_2$ (001) surface.

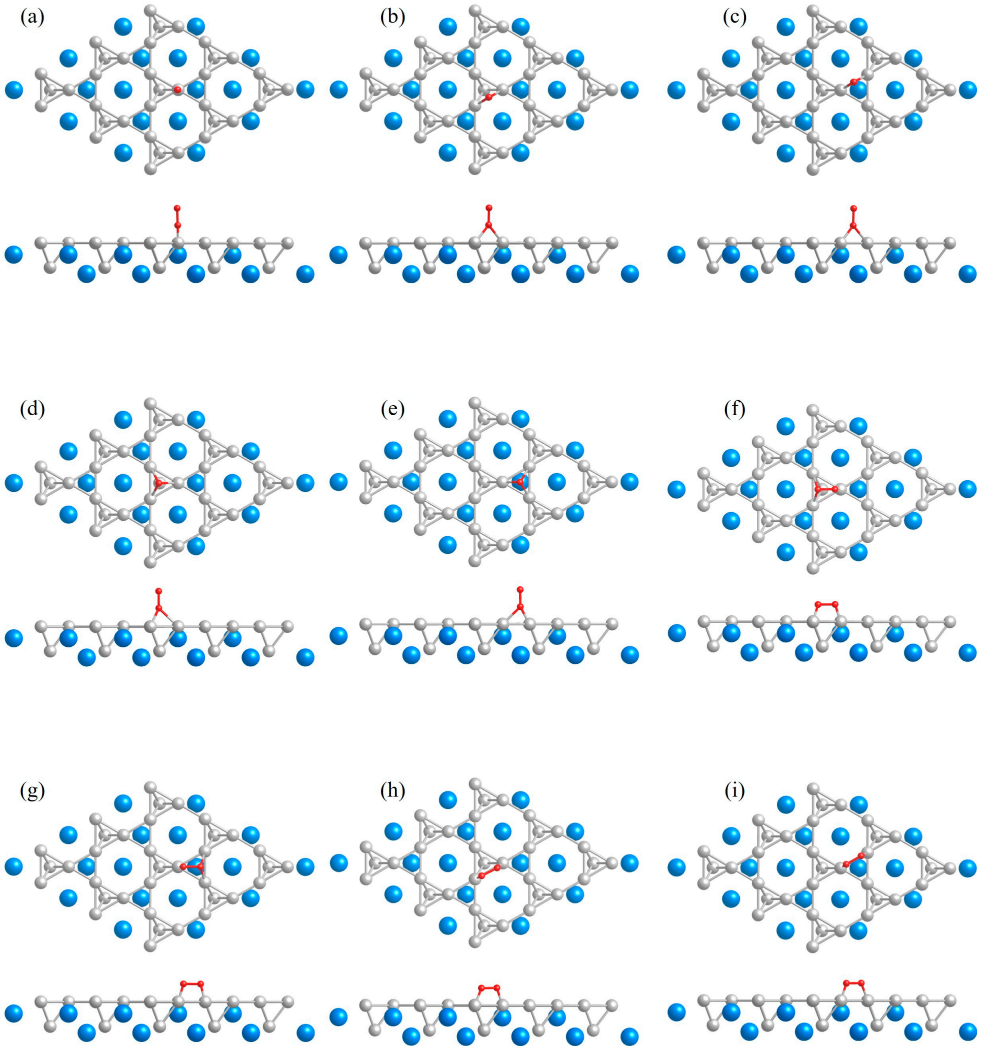

Fig. S9. Possible oxygen adsorption configurations on the catalyst. (a) to (i) represent Model #1 to Model #9.

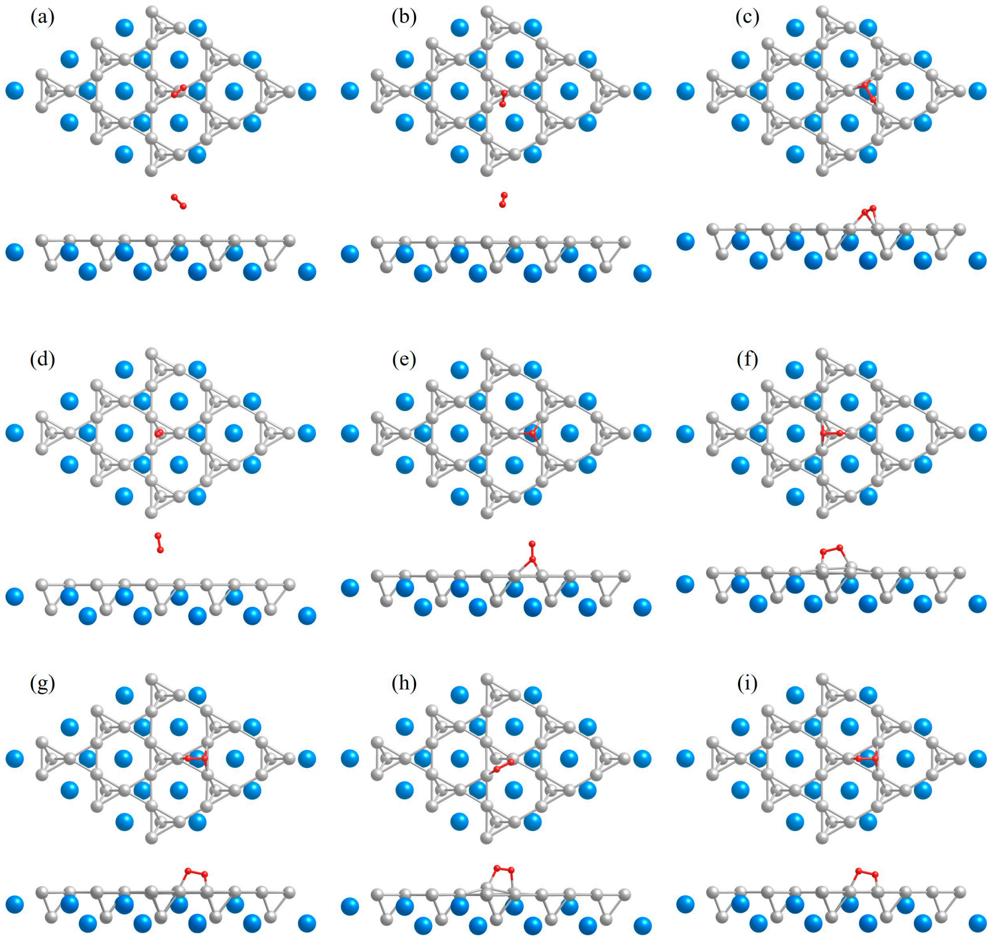

Fig. S10. Configurations after oxygen adsorption on the catalyst. (a) to (i) represent Model #1 to Model #9.

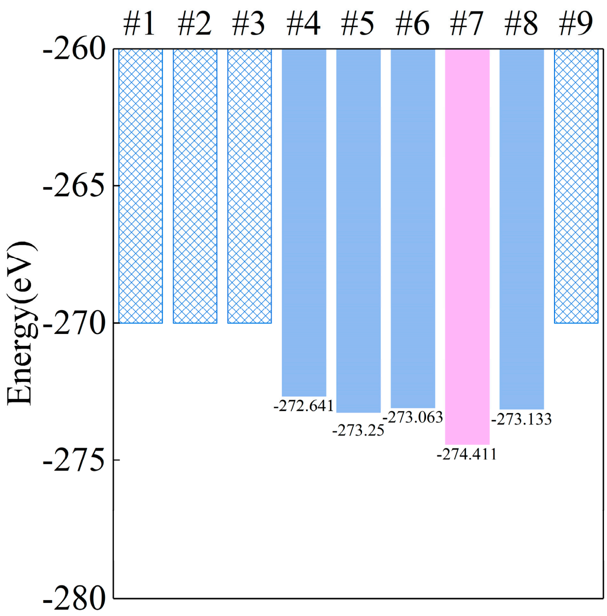

Fig. S11. Energies of different configurations of oxygen after adsorption on the catalyst.

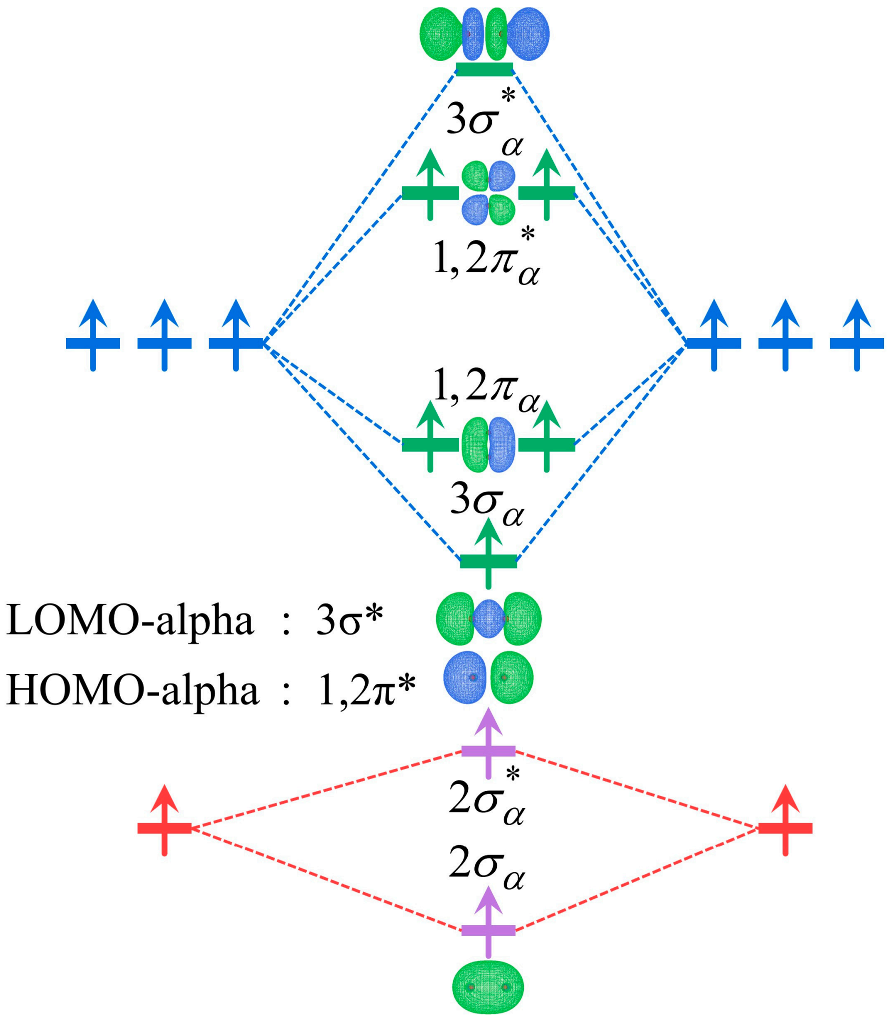

Fig. S12. Oxygen molecular orbital model (spin-up).

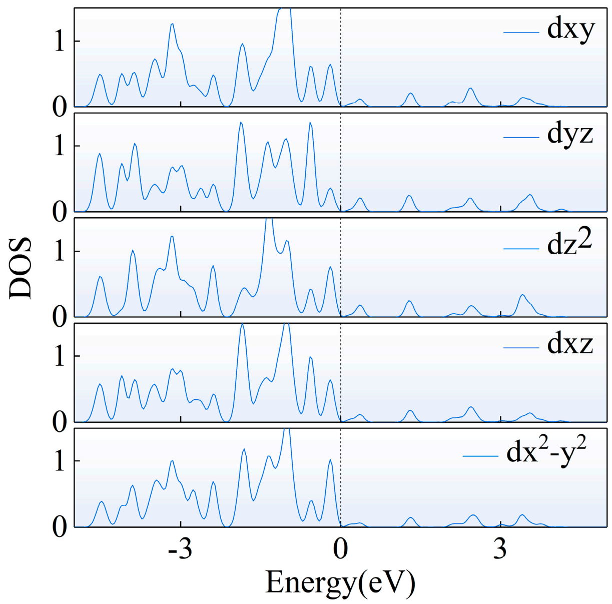

Fig. S13. Density of projected states of the d orbital (spin-up) of platinum atoms on the catalyst surface before oxygen adsorption.

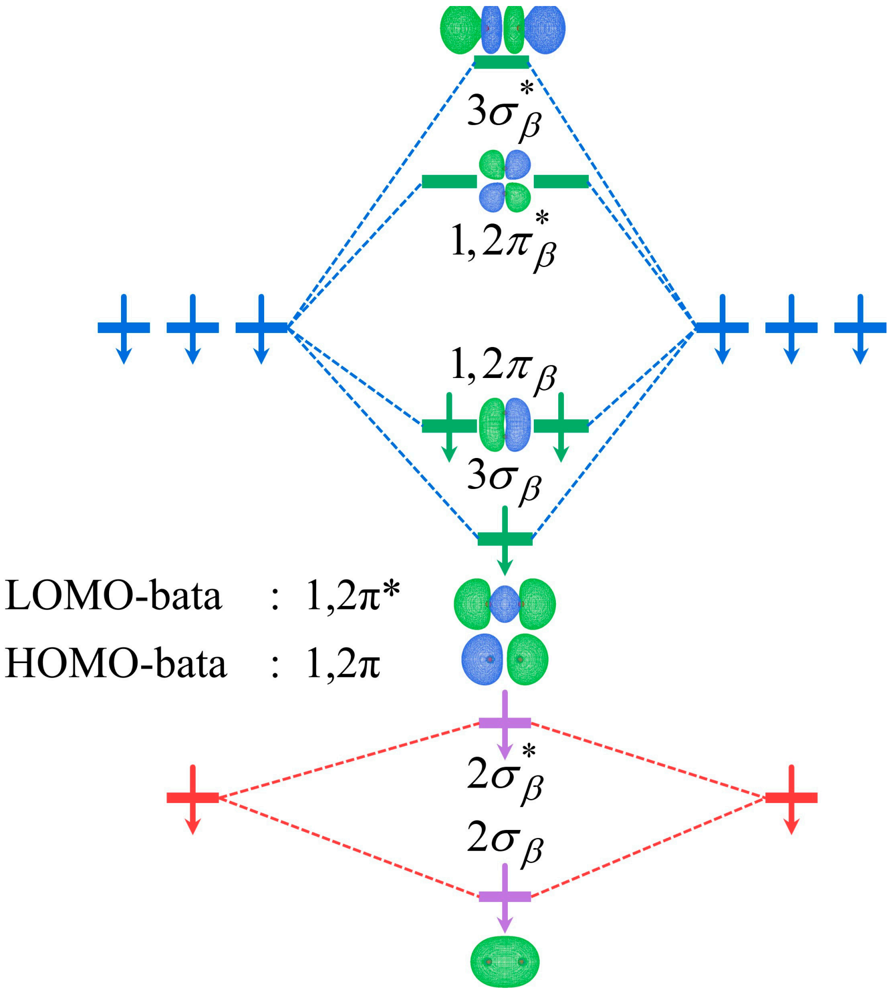

Fig. S14. Oxygen molecular orbital model (spin-down).

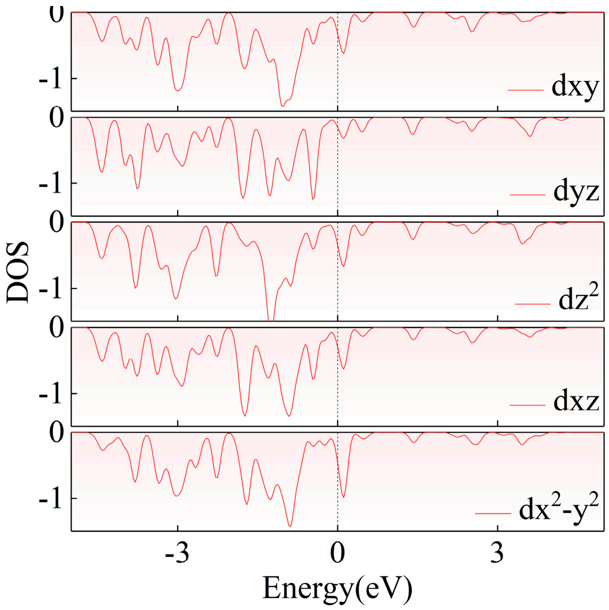

Fig. S15. Density of projected states of the d orbital (spin-down) of platinum atoms on the catalyst surface before oxygen adsorption.

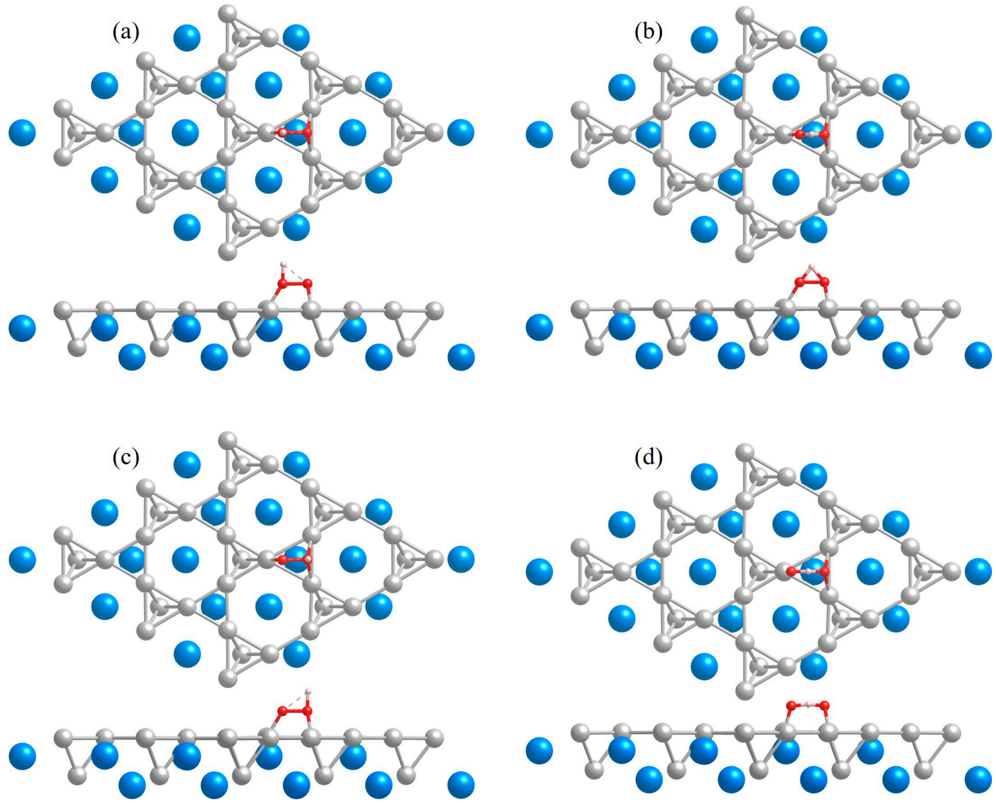

Fig. S16. Possible OOH adsorption configurations on the catalyst. (a) to (d) represent Model #1 to Model #4.

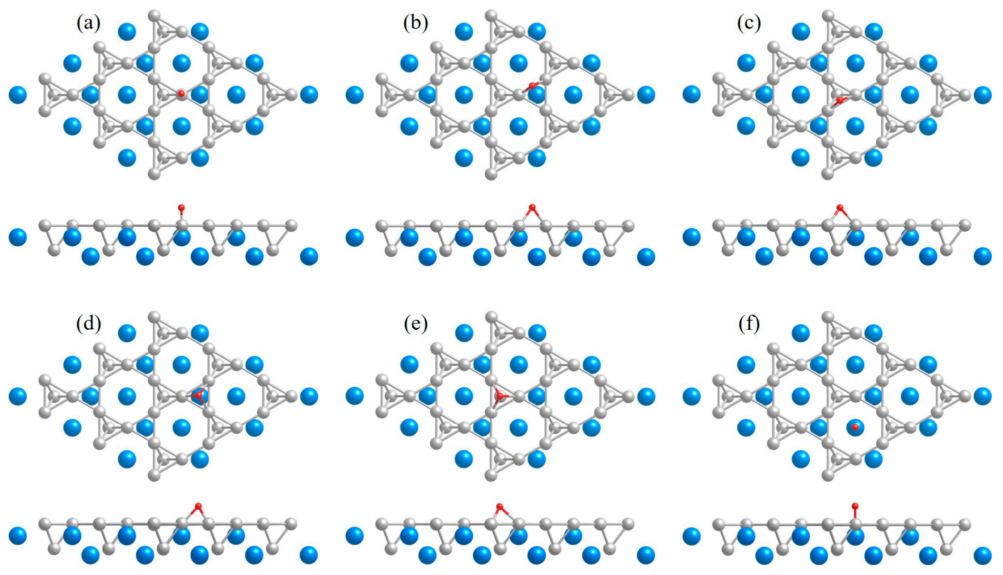

Fig. S17. Possible O adsorption configurations on the catalyst. (a) to (e) represent Model #1 to Model #6.

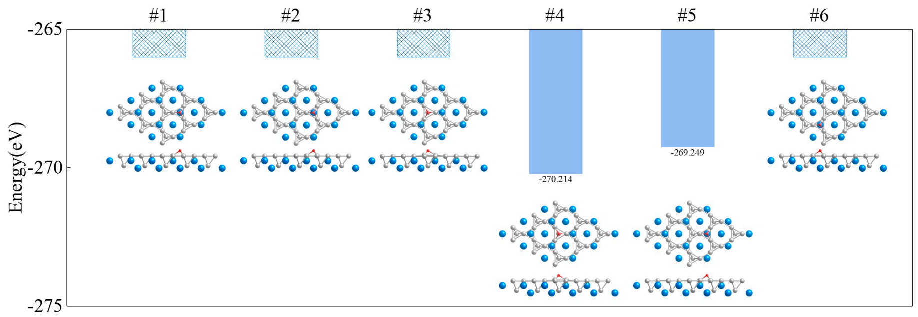

Fig. S18. Energies of the O atom adsorbed at different sites on the catalyst.

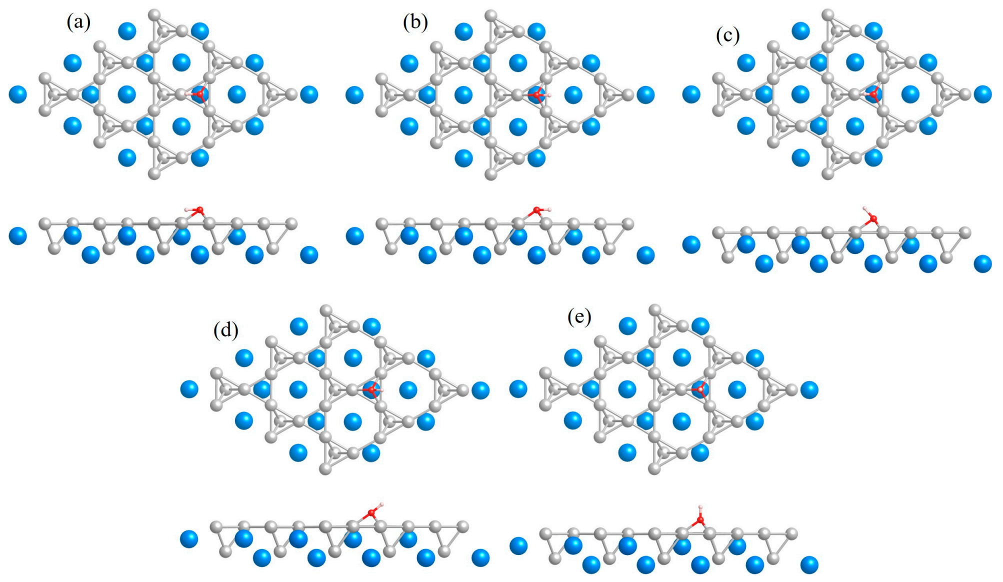

Fig. S19. Possible OH adsorption configurations on the catalyst. (a) to (e) represent Model #1 to Model #5.

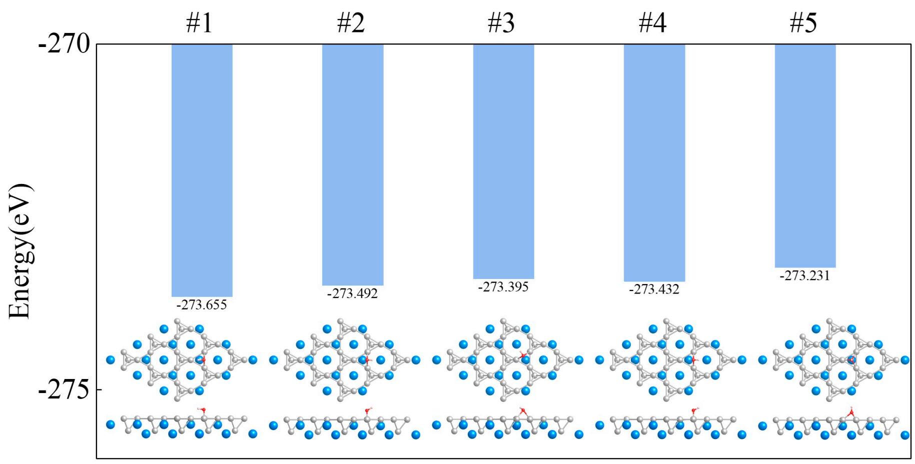

Fig. S20. Energies of different configurations of OH after adsorption on the catalyst.
